# Quasi-Steady-State Analysis based on Structural Modules and Timed Petri Net Predict System’s Dynamics: The Life Cycle of the Insulin Receptor

**DOI:** 10.3390/metabo5040766

**Published:** 2015-12-18

**Authors:** Jennifer Scheidel, Klaus Lindauer, Jörg Ackermann, Ina Koch

**Affiliations:** 1 Molecular Bioinformatics, Cluster of Excellence “Macromolecular Complexes”, Institute of Computer Science, Johann Wolfgang Goethe-University Frankfurt am Main, Robert-Mayer-Str. 11-15, 60325 Frankfurt am Main, Germany; E-Mails: J.Scheidel@bioinformatik.uni-frankfurt.de (J.S.); J.Ackermann@bioinformatik.uni-frankfurt.de (J.A.); 2 Sanofi Aventis Deutschland GmbH, 65926 Frankfurt am Main, Germany; E-Mail: Klaus.Lindauer@sanofi-aventis.com

**Keywords:** insulin receptor, Petri net, timed Petri net, transition invariant, functional modules, quasi-steady state

## Abstract

The insulin-dependent activation and recycling of the insulin receptor play an essential role in the regulation of the energy metabolism, leading to a special interest for pharmaceutical applications. Thus, the recycling of the insulin receptor has been intensively investigated, experimentally as well as theoretically. We developed a time-resolved, discrete model to describe stochastic dynamics and study the approximation of non-linear dynamics in the context of timed Petri nets. Additionally, using a graph-theoretical approach, we analyzed the structure of the regulatory system and demonstrated the close interrelation of structural network properties with the kinetic behavior. The transition invariants decomposed the model into overlapping subnetworks of various sizes, which represent basic functional modules. Moreover, we computed the quasi-steady states of these subnetworks and demonstrated that they are fundamental to understand the dynamic behavior of the system. The Petri net approach confirms the experimental results of insulin-stimulated degradation of the insulin receptor, which represents a common feature of insulin-resistant, hyperinsulinaemic states.

## 1. Introduction

Physical activity and insulin control the energy metabolism in mammalian cells. In response to elevated blood glucose levels, pancreatic beta cells located in the islets of Langerhans secrete insulin. The secreted insulin triggers the uptake of glucose in adipose and muscle tissue. Standard medical diagnosis of diabetes, insulin resistance, and other disorders of the energy metabolism include the testing of the glucose-insulin regulatory system [1,2,3], e.g., by an oral or intravenous glucose tolerance test.

**Figure 1:**
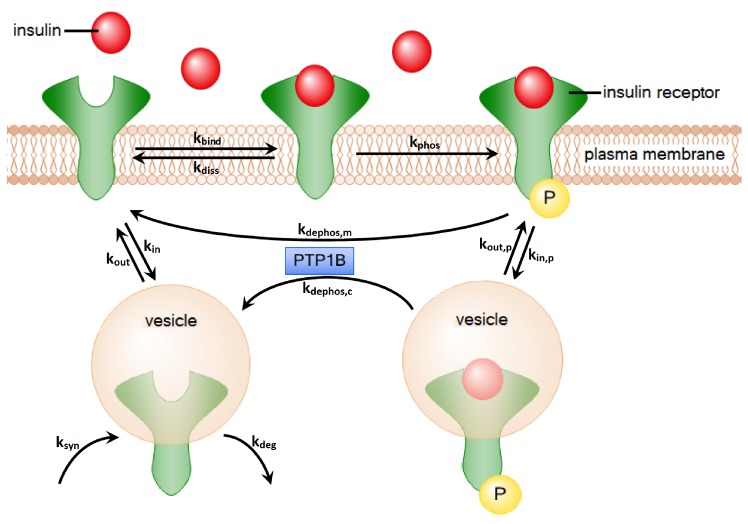
The processes of insulin-dependent activation and recycling of the IR. First, insulin binds to the IR. Afterwards, the IR gets autophosphorylated. Two alternative processes describe the dephosphorylation of the IR: (1) The IR can dephosphorylate on the plasma membrane by the dissociation of insulin; (2) The IR can be internalized into the cytoplasm. There, the insulin is degraded, and the IR is transported back to the plasma membrane. The processes of dephosphorylation on the plasma membrane and in the cytoplasm are both catalyzed by the enzyme PTP1B.

Initially, insulin binds and activates the insulin receptor (IR) located as a homodimer in the membrane of the cell. Upon a very fast binding event of insulin to the extracellular binding site of the IR homodimer [4,5], a slower rate-limiting phase occurs that manifests with a conformational change in the complex [6]. The binding of insulin to the IR triggers autophosphorylation of the intracellular IR domain, and the autophosphorylation initiates a signaling cascade for the regulation of glucose uptake [7,8]. The cell maintains the capability to tight regulation by recycling of the phosphorylated IR homodimer. The recovery of the IR is accomplished either by the dissociation of insulin from the IR or via an internalization of the entire complex (endocytosis), which moves the complex into the cytoplasm. In the cytoplasm, the insulin dissociates from the IR, and the dissociated insulin is degraded [9]. After dissociation of insulin, the IR becomes dephosphorylated and either returns back to the membrane or is degraded, see Figure 1. The recycling of the internalized IR is a critical step for the regulation of the energy metabolism and subject of intensive experimental and theoretical studies.

Since the binding of insulin and insulin analogs to the IR is of great importance to pharmaceutical applications, extensive experimental research [4,5,6,10,11,12,13,14] has been focused on this complex extracellular mechanism. The engagement of insulin with the receptor displays allosteric properties, such as negative cooperativity and insulin dependence of the dissociation rate. Several groups [15,16,17,18,19,20,21] have proposed mechanisms for the binding process. Molecular processes inside the cell are much more difficult to be experimentally accessed.

Only little is known, how the IR transmembrane domains transfer signals across the membrane and how the receptor becomes activated. A broad range of experimental methods [22,23,24,25,26] provides quantitative data, but currently, the experimental view on the molecular details of the life cycle of the IR inside the cell remains still unclear. The various theoretical models for the IR recycling do not converge on a unique, consensus network topology. The modeling of the detailed topology of the life cycle of the IR remains problematic due to many still unknown biochemical and structural properties.

**Figure 2:**
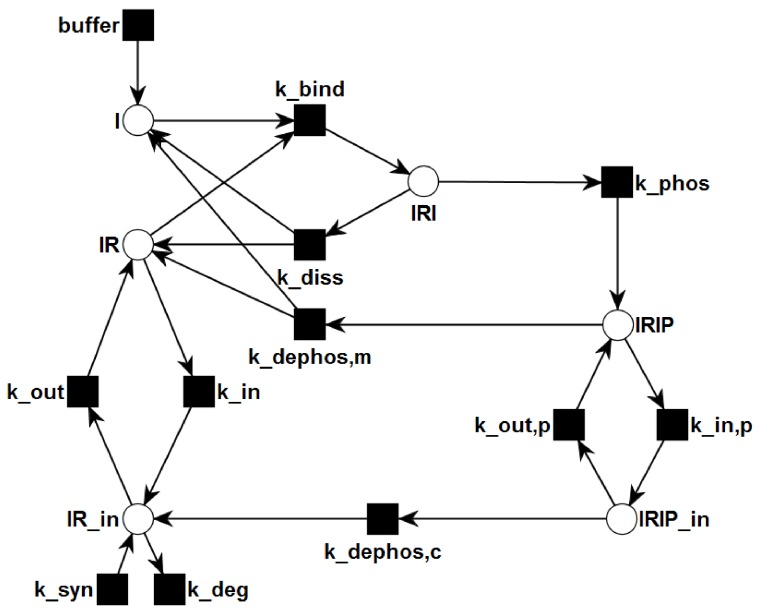
The PN describes the topological network structure of insulin dependent activation and recycling. Rectangles represent transitions, *i.e.*, reactions. Places are plotted as circles. Directed, weighted edges connect transitions and places. The places represent chemical species, e.g., insulin, receptors, or complexes, and can carry tokens, which represent discrete amounts of the chemical species. Transitions can consume tokens from the pre-places and generate tokens on the post-places.

Numerous mathematical models for the glucose-insulin regulatory system have been developed over the past 50 years; for a review see [27]. One of the first models [28,29,30] of the IR recycling is the two-component ordinary differential equation (ODE) system of Quon & Campfield [31]. Their model is able to reproduce experimental data of ligand-induced down- and up-regulation of receptors as well as the initial spontaneous display of the surface IRs for cell cultures of BC3H-1 myocytes. Extended and more detailed models have been developed and studied over the last years [10,32,33,34,35,36,37,38,39,40,41,42,43,44,45,46,47,48,49,50,51]. For a map of relationships between the models, see Figure 2 in the review of Ajmera *et al.* [52]. Each proposed model is able to reproduce a given set of experimental data measured under specific conditions for a given cell type, e.g., adipocytes, lymphocytes, hepatocytes, or myocytes. The values for the kinetic and equilibrium rate constants depend on several factors, such as cell type, temperature, and composition of the culture media.

The IR is synthesized in the endoplasmic reticulum and delivered via the Golgi apparatus to the plasma membrane. Ligand binding triggers endocytosis, and the internalized activated tyrosine kinase receptor is a target for effective lysosomal degradation [53,54]. The main focus of mathematical modeling is the regulation of glucose uptake within the time range of minutes up to few hours. Consequently, the models either completely neglect the synthesis and degradation of the IR or apply a reaction rate constant of $k=1.67\times 10^{-18}$ min${}^{-1}$ [34,35,41,42,51] for the degradation process. This assumes an astronomic time scale of $1.1\times 10^{12}$ years, leading to a theoretical steady-state value of 100 M IR concentration for a cell [37]. Recently, Song *et al.* [55] have demonstrated that the E3 ligase activity of the muscle-specific mitsugumin 53 (MG53) regulates the IR stability through ubiquitin-dependent degradation. They have identified the protein, MG53, as a therapeutic target for treating metabolic disorders. Note that the down-regulation of the IR by insulin-stimulated endocytosis and degradation is a common feature of most insulin-resistant, hyperinsulinaemic states [56,57,58,59,60].

This work presents a Petri net (PN) approach [61,62,63,64,110] for the modeling of the IR recycling. PNs are worthwhile for modeling the IR recycling for two reasons. First, all theoretical models published so far in the literature are based on the approximation of the kinetics by the mass action principle. Conventionally, a mass action principle formulates an ODE system [65,66,67] or a master equation [68,69,70] for discrete entities. The mass action principle assumes an isotropic and homogeneous physical environment in which a molecule can freely move in any direction, which is not given for the movement of the IR in the environment of a highly structured and organized cell. Second, the steps involved in the IR recycling are specific to the cell type and the environmental conditions of the cell. A model may approximate the system’s behavior for given experimental conditions, but the application to another cell type or to altered environmental conditions at least requires the adaption of the model, e.g., by refitting the rate constants and initial concentrations.

We chose the network topology, shown in Figure 2, in accordance with the reaction system of Sedaghat *et al.* [34], who proposed a mathematical model of the metabolic insulin signaling pathways. They abstain from describing allosteric properties of the insulin binding mechanism. Their model gives a coarse grain, but consistent description of the reactions. In contrast to the majority of more complex models, this model describes a complete life cycle of the receptor, including the steps of synthesis and degradation. Despite its simplicity the model reflects the basic mechanisms well. It has sufficient variability to approximate the kinetics of different cell types for a broad range of experimental conditions if the rate constants are appropriately refitted. The properties of the model have been thoroughly studied [37,38,42,51,71]. Despite its limitations [46] the model provides a simple reference network topology which can be easily extended.

In this paper, we present a PN model for the IR activation and recycling. We focused on molecular processes that regulate the response of a cell to an alteration of the level of secreted insulin. We use the PN formalism, because it is independent of the physical concept and is able to provide ways to explore concepts, alternative to the mass action kinetics. The PN approach analyzes the structure of a system of interacting entities. Neither the values of the rate constants nor the detailed kinetics of each reaction have to be known a priori. The basic prerequisite for a PN approach is the knowledge of the topology of the IR life cycle.

The paper is organized as follows. In the first step, we briefly give the main definitions and the properties of PNs and timed PNs (TPNs). Then, we describe the PN and TPN for the IR system. TPNs employ special time-dependent firing rules to generate the dynamics of token numbers that may be capable of mimicking the IR life cycle. In the next part, we apply standard structural PN analysis to the network topology. We focus on transition invariants (TIs) and their interpretation to understand the functional modules of the IR life cycle. Based on the TI, we derive analytical expressions for critical parameters and the time course of concentrations of mass action reaction kinetics. The results of the PN approach demonstrate that, even without knowing the kinetic parameters, the experimental findings of insulin-induced long-term down-regulation of the cellular IR level can be confirmed [53,72,73,74,75,76,77,78,79,80,81,82,83,84,85,86,87,88,89,90,91].

## 2. Methods

### 2.1. Petri Nets

Petri nets are directed, bipartite graphs. The two types of vertices called *places* and *transitions* define the passive and the active components, respectively, of the system. Vertices are connected by directed, labeled edges in such a way that only edges between vertices of different type exist. Considering a transition, the places connected by edges, pointing to the transition, are called *pre-places*, and places connected by edges, coming from the transition, are called *post-places*. Considering a place, *pre-transitions* and *post-transitions* are defined analogously.

*Definition Petri net (PN): A PN is a* 5*-tuple $PN=(P,T,F,W,M_{0})$, where*

- P is a finite set of places,
- T is a finite set of transitions,
- F⊆(P×T)∪(P×T) is a set of edges,
- W:F→N is the set of edge weights, and
- $M_{0}:P\to N_{0}$ is the initial marking.

The dynamics of the system is implemented using movable objects called *tokens* which are located on the places. A certain token distribution defines a certain system state. Tokens can move from one place to another one via transitions, following a *firing rule*. First, the transition has to be activated, *i.e.*, its pre-places have to carry at least as many tokens as indicated by the corresponding edge weights. In *Place/Transition-PNs* (P/T-PNs), tokens will be consumed and removed at the same time. So, the firing rule does not consider any time constraints or parameters. For a detailed introduction into PN formalism see [61,62,64,110], and for their application in biology, see [63,107,111].

#### 2.1.1. Timed Petri Nets

TPNs explicitly consider the time, for example as time stamps of tokens or time delay of transitions. Several definitions are possible, see [92] for a formal introduction. Simulating a TPN, the firing rule uses a global clock time and follows an artificial integer simulation time (clock) that should not be confused with the physical reaction time. The global clock time is initialized with zero before starting the simulation.

We apply the concept of colored PNs (CPNs) [93] to represent a TPN, using the software *CPN Tools* [93,94]. CPN Tools provides the opportunity to *color* a timed token by an associated number called *time stamp*. At a given clock time, a timed token is either active, if its time stamp is less than or equals the global clock time or inactive, otherwise. In a TPN, a transition is capable of processing an infinitely large number of tokens at a given clock time. We characterize a transition additionally by a constant delay expression of a transition time inscription.

The firing rule can be described as follows. A transition is active if a sufficient number of active tokens is available on its pre-places. The algorithm chooses randomly one transition from the set of active transitions. The selected transition fires by removing active tokens from the pre-places and creating tokens on the post-places. The time stamp of a token created on a post-place is the current global clock time plus the time delay of the firing transition. The random selection and firing of active transitions continues until the set of active transitions is empty. When the set of active transitions is empty and no further transition can fire, the algorithm increments the clock time until at least one transition is enabled. To limit the maximal firing rate of a transition, we complemented each transition by a place called *time generation place* (TGP). A TGP is a pre- and post-place of a transition. It carries a single timed token which is necessary to activate the transition, but will not be consumed by firing of the transition. At every time point the transition fires, the time stamp of the token increases and the single token of the TGP becomes inactive.

**Figure 3:**
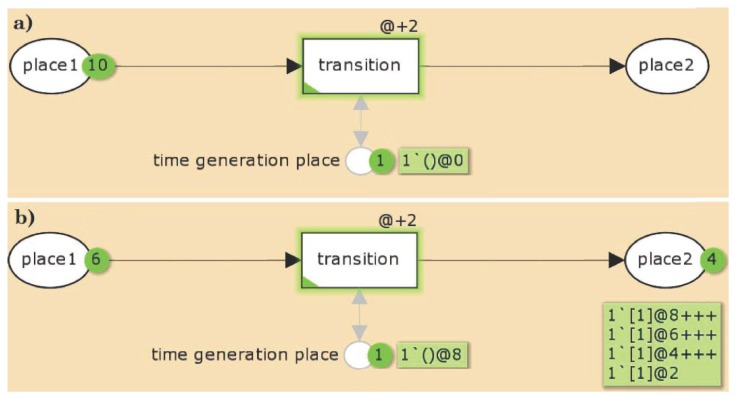
An example for a TPN. The TPN consists of three places, *place 1*, *place 2*, and a time generation place (TGP), and one transition connected by directed edges with an edge of weight 1. (**a**) In the initial state of the TPN, *place 2* carries no token and *place 1* 10 tokens, each of them with a time stamp of @0. There exists a global clock time (not explicitly depicted) which is in the initial state 0. For the transition, a time delay of @ + 2 is defined, indicating that the time stamp of each token processed by this transition increases by 2. The TGP is a pre- and post-place of the transition, *i.e.*, the token on that place will not be consumed when the transition fires. This token is initialized with a time stamp of @0. ($1^{\prime }\left(\right)$ and $1^{\prime }\left[1\right]$ are specific notations of the CPN Tools.); (**b**) The state of the TPN after four simulation steps. 4 tokens on *place 1* were consumed and produced on *place 2*, carrying the time stamps, @2,@4,@6, and @8, respectively. The other six tokens remain still on *place 1* with a time stamp of zero. The transition is enabled if on *place 1* and the TGP exist at least one token with a time stamp less or equals the global clock time. If there is no transition activated anymore, the global clock time will be increased until at least one transition can fire. Here, the global clock time has to be increased by 2 with each firing step. The TGP ensures that the tokens can be distinguished from each other, *i.e.*, only one token is moved from *place 1* to *place 2* for a given global clock time. After the first firing step, the time stamp of the TGP token and the token on *place 2* have a time stamp of 2. The global clock time is still 0. Now, the transition is not anymore enabled, because there is no token with a time stamp less than or equals the global clock time on the TGP. For the next firing, the global clock time has to be increased by 2.

Figure 3 illustrates a small example of a TPN. Figure 3a depicts the TPN before the simulation. The TPN consists of three places, *place 1*, *place 2*, and a TGP, and one transition connected by directed edges with an edge of weight 1. In the initial state of the TPN, *place 2* carries no token and *place 1* 10 tokens, each of them with a time stamp of @0. There exists a global clock time (not explicitly depicted) which is in the initial state, *i.e.*, equals 0. For the transition, a time delay of @ + 2 is defined, indicating that the time stamp of each token processed by this transition increases by 2. The TGP is a pre- and post-place of the transition, *i.e.*, the token on that place will not be consumed when the transition fires. This token is initialized with a time stamp of @0.

Figure 3b depicts the state of the TPN after four simulation steps. 4 tokens on *place 1* were consumed and produced on *place 2*, carrying the time stamps, @2,@4,@6, and @8, respectively. The other six tokens remain still on *place 1* with a time stamp of zero. The transition is enabled if on *place 1* and on the TGP exist at least one token with a time stamp less or equals the global clock time. If there is no transition anymore activated, the clock time will be increased until at least one transition can fire. Here, the global clock time has to be increased by 2 with each firing step. The TGP ensures that the tokens can be distinguished from each other, *i.e.*, only one token is moved from *place 1* to *place 2* for a given global clock time. After the first firing step, the time stamp of the TGP token and the token on *place 2* have a time stamp of 2. The global clock time is still 0. Now, the transition is not anymore enabled, because there is no token with a time stamp less than or equals the global clock time on the time generation place. For the next firing, the global clock time has to be increased by 2.

#### 2.1.2. General Properties

Several graph-based properties can be defined for PNs. Here, we consider those that have a relevance in modeling biochemical networks. For a more detailed overview, see [95]. A net is *pure* if it is loop-free, *i.e.*, there exist no transition, for which a pre-place is also a post-place. A biological PN is not pure, if they explicitly model, for example, catalytic reactions, using *read* or *test edges*, *i.e.*, loops. A PN is *ordinary*, if every edge weight equals one. This is often valid for signal transduction networks, but rare for metabolic systems, where we often have stoichiometric numbers greater than one. *Homogeneity* describes the property for any place, when all edges, starting from the considered place, have the same weights. A network model should be *connected*, *i.e.*, there exist a undirected path from each vertex to each other vertex. An important property is *liveness*, meaning that there is no deadlock in an initial marking. A PN is *live*, if all its transitions are live in the initial marking, *i.e.*, no state is reachable in which a transition is dead, meaning, it can never fire again. Liveness is a useful property in particular during the modeling process. Simulating the network, dead locks will easily be indicated. But, for being sure that there is no deadlock, liveness has to be checked.

#### 2.1.3. Invariant Properties

Invariant properties of a network are important because they describe the complete basic systems behavior. They can be used for network verification regarding completeness and correctness of a network. In PNs, two types of invariants can be defined, the *place invariants*, leading to substance conservation rules, and the *transition invariants*, giving functionally basic subnetworks.

The definitions of the invariants are based on the *incidence matrix*. The incidence matrix *C* of a PN is an (n×m)-matrix, where *n* denotes the number of places and *m* the number of transitions. Every matrix entry $c_{ij}$ gives the token change on the place $p_{i}$ by the firing of the transition $t_{j}$. A transition invariant (TI) is defined as a non-zero vector $x\in N_{0}^{m}$ which holds the equation

(1) C·x=0

A TI represents a multiset of transitions whose firing has no effect on the marking, *i.e.*, if all of them have fired the required number of times, an arbitrary initial marking is reproduced. A *trivial* TI consists of one forward and one backward reaction. Verifying a PN, the non-trivial TIs are of interest. For the correctness, each of them should have a senseful biological interpretation. A net holds the *CTI* property or it is *Covered by TIs*, respectively, if each transitions of the PN belongs to at least one TI. A transition which is not a member of one TI could be removed, because it is not involved in system’s dynamics. Thus, the CTI property represents a completeness criterion. The transitions of a TI with all the places and edges in between defines a subnetwork which represents a certain function of the system and can be interpreted as a functional module. These subnetworks can overlap.

Analogously, a place invariant (PI) is defined as a non-zero vector $y\in N_{0}^{n}$ which holds the equation

(2) y·C=0

A PI characterizes a token conservation rule for a set of places, over which the weighted sum of tokens is constant independently from any firing, *i.e.*, for a PI *y* and any markings $m_{i},m_{j}\in N_{0}^{n}$, which are reachable from $M_{0}$ by the firing of transitions, it holds

(3) $$y\cdot m_{i}=y\cdot m_{j}$$

The non-zero entries of an invariant *x*, are called its support written as supp(x). An invariant *x* is called *minimal*, if its support does not contain the support of any other invariant *z*, *i.e.*,

(4) $$\begin{array}{c} ∄\;\text{invariant }z:supp\;(z)\subset supp\;(x) \end{array}$$

and the greatest common divisor of all non-zero entries of *x* is one. In the following, we consider minimal, non-trivial TIs and PIs. We applied the open-source software MonaLisa [96,97,109].

## 3. Results and Discussion

Both PN models, the P/T model and the TPN model, are based on the reaction system proposed by Sedaghat *et al.* [34]. For the lists of abbreviations see Table 1 and Table 2.

(5) $$\begin{array}{ccccccc} \text{IR}+\text{I} & \overset{k_{\text{bind}}}{\underset{k_{\text{diss}}}{⟺}} & \text{IRI} & \overset{k_{\text{phos}}}{⟶} & \text{IRIP} & \overset{k_{\text{dephos,m}}}{⟶} & \text{IR}+\text{I}\\ \text{IRIP} & \overset{k_{\text{in,p}}}{\underset{k_{\text{out,p}}}{⟺}} & {\text{IRIP}}_{in} & \overset{k_{\text{dephos,c}}}{⟶} & {\text{IR}}_{in} & \overset{k_{\text{out}}}{\underset{k_{\text{in}}}{⟺}} & \text{IR}\\ \emptyset & \overset{k_{\text{syn}}}{\underset{k_{\text{deg}}}{⟺}} & {\text{IR}}_{in} & & & \end{array}$$

**Table 1:** List of abbreviations and initial concentrations. Initial concentrations are adopted from Sedaghat *et al.* [34]. The concentrations are given in units of pM $=10^{-12}\;$M.

Abbreviation	Species	Initial Concentration(s) [pM]
I	insulin	[I]${}_{0}=10^{3}$–$10^{6}$
IR	insulin receptor	[IR]${}_{0}=0.9$
IRI	I-IR complex	—
IRIP	phosphorylated IRI	—
IRIP${}_{in}$	intracellular IRIP	—
IR${}_{in}$	intracellular IR	[IR${}_{in}$]${}_{0}=0.1$

**Table 2:** List of kinetic parameters of the reaction system (5). The kinetic parameters are adopted from Sedaghat *et al.* [34]. For the rates of degradation and synthesis of the IR in the cytoplasm, we chose the values of Quon and Campfield [31].

Parameter	Process	Value	Units
$k_{\text{bind}}$	binding of insulin	$6\times 10^{7}$	M${}^{-1}$ min${}^{-1}$
$k_{\text{diss}}$	dissociation of insulin	0.2	min${}^{-1}$
$k_{\text{phos}}$	phosphorylation	2.500	min${}^{-1}$
$k_{\text{dephos,m}}$	dephosphorylation on membrane	0.2	min${}^{-1}$
$k_{\text{in}}$	internalization of IR	$3.\bar{3}\times 10^{-4}$	min${}^{-1}$
$k_{\text{out}}$	transport of IR to plasma membrane	$3\times 10^{-3}$	min${}^{-1}$
$k_{\text{in,p}}$	internalization of phosphorylated IR	$2.1\times 10^{-3}$	min${}^{-1}$
$k_{\text{out,p}}$	transport of phosphorylated IR to plasma membrane	$2.1\times 10^{-4}$	min${}^{-1}$
$k_{\text{dephos,c}}$	dephosphorylation in cytoplasm	0.461	min${}^{-1}$
$k_{\text{deg}}$	degradation	$1.67\times 10^{-4}$	min${}^{-1}$
$k_{\text{syn}}$	synthesis	${}^{*1}$ $1.67\times 10^{-17}$	M min${}^{-1}$
		${}^{*2}$ $1.00\times 10^{-16}$	M min${}^{-1}$

${}^{*1}$ if IR${}_{in}\geq 10^{-13}$ M; ${}^{*2}$ if IR${}_{in}<10^{-13}$ M.

### 3.1. The P/T-PN Model and Its Properties

Figure 2 depicts the PN model of the reaction system Equation (5). The six places drawn as circles represent the species I, IR, IRI, IRIP, IR${}_{in}$, and IRIP${}_{in}$, and the ten transitions drawn as filled squares describe the changes of species. Since insulin is supplied by the environment to the system, the species I is defined to be *external*. External metabolites can be produced or consumed arbitrarily. The transition *buffer* causes the external production of insulin. In terms of PN formalism [95] the network is *pure*, *ordinary*, *homogenous*, *connected*, and *live*.

*Place Invariants:* The PN has no PI because the model includes an adaption of the cell to a variation of external insulin concentration via synthesis and degradation of the IR. Neglecting the synthesis and degradation of the IR, the places IR, IRI, IRIP, IR${}_{in}$, and IRIP which represent the IR in its various forms, e.g., extracellular, internalized, phosphorylated, would be members of a PI. Such a PI would account for a conservation of the total amount of IR in the cell. But, due to the synthesis and degradation of the IR, the cell can adapt its total amount of receptor to an increased concentration of external insulin.

**Figure 4:**
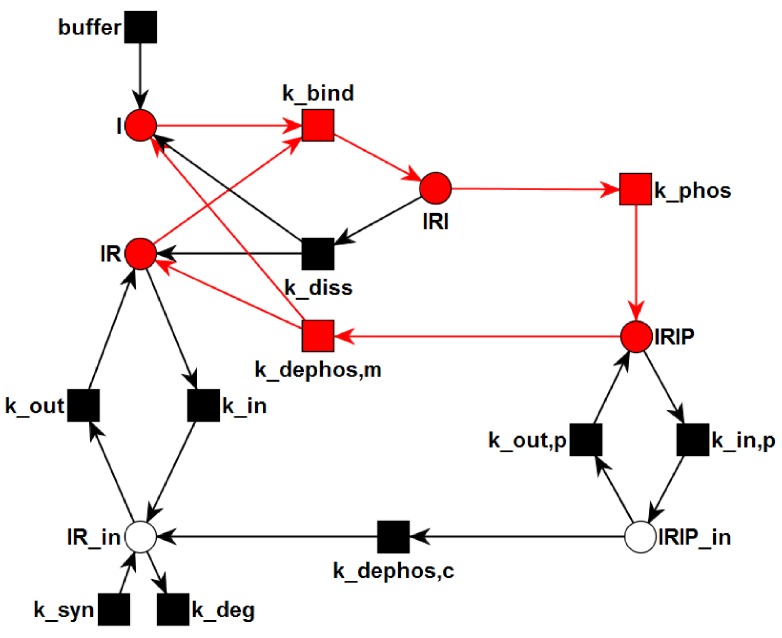
The transition invariant, TI${}_{1}$, is highlighted in the PN. TI${}_{1}$ describes a cycle of the processes: binding of insulin to the IR ($k_{\text{bind}}$), phosphorylation of the insulin-IR complex ($k_{\text{phos}}$), and extracellular dissociation of the activated insulin-IR complex ($k_{\text{dephos,m}}$). The steady state of this subnetwork has an equilibrium constant of $i_{c}=3.33$ nM for the binding of insulin to the receptor.

**Figure 5:**
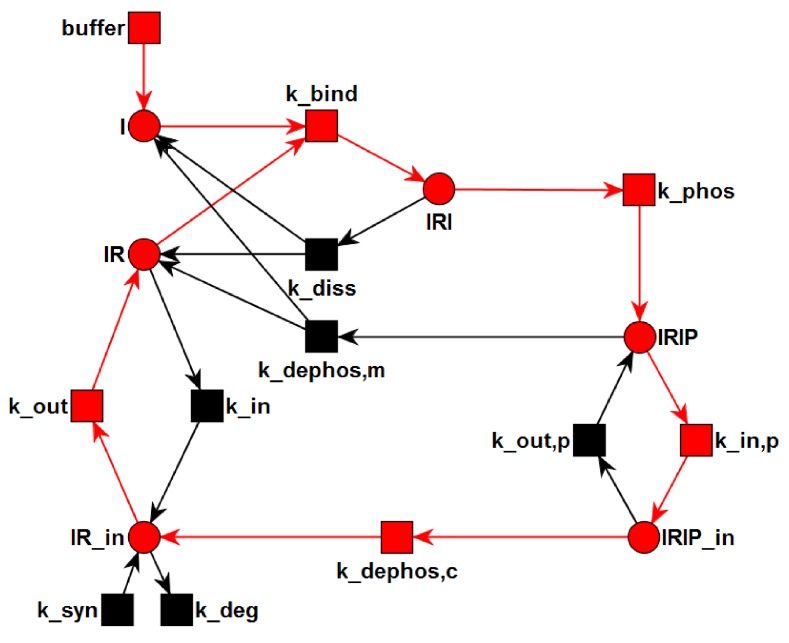
The transition invariant, TI${}_{2}$, is highlighted in the PN. The six transitions of TI${}_{2}$ form a chain of six consecutive reactions: buffering of insulin (*buffer*), binding of insulin to the IR ($k_{\text{bind}}$), phosphorylation of the insulin-IR complex ($k_{\text{phos}}$), internalization of the activated insulin-IR complex ($k_{\text{in,p}}$), dephosphorylation of the internalized insulin-IR complex ($k_{\text{dephos,c}}$), and translocation of internalized IR back to the membrane ($k_{\text{out}}$).

*Transition Invariants:* The network has six TIs, see Supplemental Material for a list of the TI. Two invariants, TI${}_{1}$ and TI${}_{2}$, are non-trivial. TI${}_{1}$ highlighted in Figure 4 is a cycle of the processes: binding of insulin to the IR ($k_{\text{bind}}$), phosphorylation of the insulin-IR complex ($k_{\text{phos}}$), and extracellular dissociation of the activated insulin-IR complex ($k_{\text{dephos,m}}$). Note that the extracellular recycling of the IR given by the chain of the elementary (equimolar) reactions, $k_{\text{bind}}$ and $k_{\text{phos}}$; and $k_{\text{dephos,m}}$ does not alter the total concentration of insulin, *i.e.*, i+iri+irip= constant, or the total concentration of receptor, *i.e.*, ir+iri+irip= constant. TI${}_{2}$ highlighted in Figure 5 describes a chain of 6 reactions: buffering of insulin (*buffer*), binding of insulin to the IR ($k_{\text{bind}}$), phosphorylation of the insulin-IR complex ($k_{\text{phos}}$), internalization of the activated insulin-IR complex ($k_{\text{in,p}}$), dephosphorylation of the internalized insulin-IR complex ($k_{\text{dephos,c}}$), and translocation of internalized IR back to the membrane ($k_{\text{out}}$).

### 3.2. The TPN Model and Its Properties

**Figure 6:**
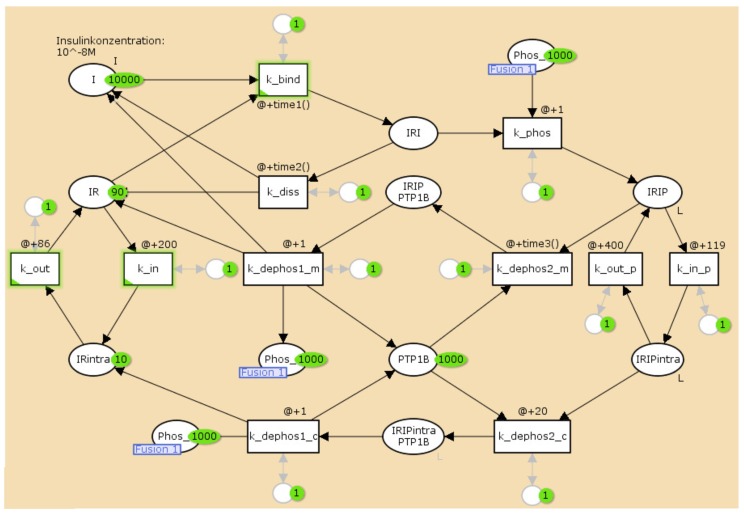
The TPN model of activation and recycling of the IR. It explicitly includes the enzyme, *protein-tyrosine phosphatase 1B* (PTP1B), see Figure 1 for a sketch of the catalytic function of PTPN1B. The circles represent chemical species, here, mainly the different insulin complexes, see Table 3. The rectangles describe the transitions, see Table 4. For a full list of places and transitions of the TPN, we refer to Table 3 and Table 4, respectively.

For the TPN model, we wanted to generate a time behavior that is similar to the numerical solution of the mass action reaction system of Sedaghat *et al.* [34]. Since the competition of the two binding sites of the receptor is not modeled quantitatively by Sedaghat *et al.*, we neglected allosteric effects and considered the ligand-receptor affinity for the binding of only one insulin molecule. Figure 6 outlines the TPN model for the IR life cycle. For the lists of abbreviations and kinetic constants, see Table 1 and Table 2, respectively. The TPN explicitly models the enzyme *protein-tyrosine phosphatase 1B* (PTPN1B), see Figure 1 for a sketch of the catalytic function of PTPN1B. For a full list of places and transitions of the TPN, we refer to Table 3 and Table 4, respectively.

**Table 3:** List of places of the TPN and the initial numbers of tokens.

Name	Molecule	Initial Number of Tokens
I	insulin	10,000
IR	insulin receptor	90
IRI	I–IR complex	0
IRIP	phosphorylated IRI	0
IRIP${}_{intra}$	intracellular IRIP	0
IR${}_{intra}$	intracellular IR	10
PTPN1B	protein-tyrosine phosphatase 1B	1000
IRIP PTP1B	IRIP–PTPN1B complex	0
IRIP${}_{intra}$ PTPN1B	IRIP${}_{intra}$–PTPN1B complex	0
Phos	phosphate	1000

**Table 4:** List of transitions of the TPN model of receptor phosphorylation and recycling. The time inscriptions of the transitions are constant delay increments. We adapted the values of the constant delays to each initial insulin concentration of 1μM, 100nM, 10  nM, and 10nM separately. This list exemplifies the constant delays for $i_{0}=1\;\mu$M.

Name	Process	Time Inscription
bin_1	binding of insulin	@ + 1
dis_1	dissociation of insulin	@ + 40
autophos_1	phosphorylation of IRI	@ + 1
intra_1	internalization of IR	@ + 200
memb_1	transport of IR${}_{intra}$ to plasma membrane	@ + 85
intra_2	internalization of IRIP	@ + 110
memb_2	transport of IRIP${}_{intra}$ to plasma membrane	@ + 400
dephos_1	dephosphorylation of IRIP by PTPN1B	@ + 1
dephos_2	IRIP binds to PTPN1B	@ + 40
dephos_3	IRIP${}_{intra}$ binds to PTPN1B	@ + 20
dephos_4	dephosphorylation of IRIP${}_{intra}$ by PTPN1B	@ + 1

**Figure 7:**
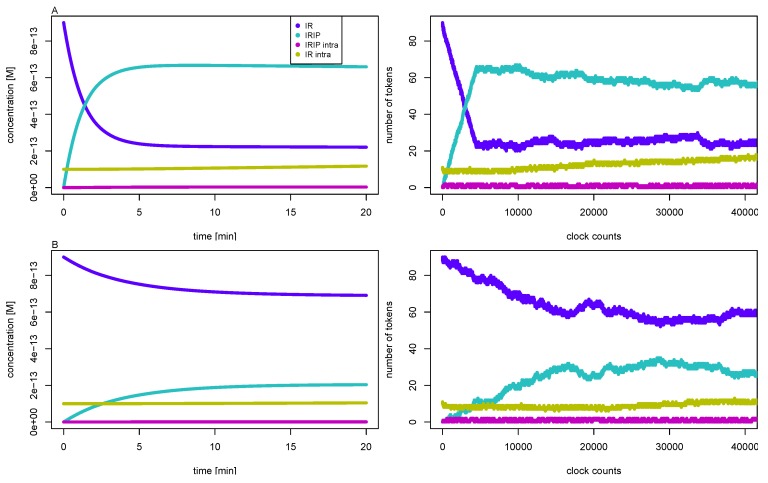
The left part depicts the concentrations of the IR (dark blue line), the phosphorylated insulin-IR complex IRIP (light blue line), the internalized complex IRIP${}_{intra}$ (purple line), and the intracellular IR${}_{intra}$ (yellow line) versus reaction time for initial insulin concentrations, 10 nM (part A, top), and 1 nM (part B, bottom), respectively. The concentrations are precise numerical simulations of the reaction system Equation (5). The right part shows the number of tokens versus the global clock time for the corresponding TPN. One token equates to a concentration of about 10 fM, and 2000 counts of the global clock time represent one minute reaction time.

It turned out that we had to manually adapt the time inscriptions individually for each initial concentration of insulin to mimic the expected time course of concentrations. Table 5 lists the appropriate constant time delays for the simulation of 1 *μ*M, 100 nM, 10 nM, and 1 nM initial insulin concentrations. Figure 7 shows the simulation results for 10 nM (upper part A) and 1 nM (lower part B) initial concentrations of insulin, respectively. The right part depicts the time course of token numbers that resemble the concentration curves of the corresponding mass action reaction systems shown in the left part. For low insulin concentrations of 10 nM and 1 nM, the IR never becomes completely saturated, a fraction of the receptor remains accessible to insulin, and the concentration of free receptor approaches a non-zero steady-state concentration. Such steady states are of crucial importance for the analysis of the ODE model, but the concept of a stable and attractive steady state can not be transferred to a TPN model. The reason is that the maximal firing rate of a timed transition is independent from the occupancy of tokens on its pre-places. As a result, the net production of tokens on a place would be either positive or negative. No state is possible at which the firing rates of active transitions counterbalance to a zero net production of tokens. Consequently, in the course of simulating the TPN model, we adjusted the time delays of the transitions, see Table 5. For such dynamically adapted time delays, the time courses of token numbers show a visual resemblance to the concentration curves, see Figure 7.

**Table 5:** Adaption of time delays of timed transitions to initial concentrations of insulin, 1 *μ*M, 100 nM, 10 nM, and 1 nM. The values in parentheses are applied after 4500 and 15,000 clock times for the insulin concentrations, $i_{0}=10$ nM and $i_{0}=1$ nM, respectively.

Transition	1 *μ*M	100 nM	10 nM	1 nM
bin_1	@ + 1	@ + 7	@ + 16 (@ + 29)	@ + 20 (@ + 30)
dis_1	@ + 40	@ + 40	@ + 40 (@ + 39)	@ + 40 (@ + 60)
memb_1	@ + 85	@ + 85	@ + 86	@ + 86
intra_2	@ + 110	@ + 110	@ + 119	@ + 119
dephos_2	@ + 40	@ + 40	@ + 40 (@ + 52)	@ + 40 (@ + 65)

### 3.3. Quasi-Steady-State Approximation

The time evolution of the insulin system is conventionally studied for the response of the cell to a high (typically 4-6 orders of magnitude larger than that of the IR), external insulin concentration, or *vice versa* for the regulation of the cell back to its basal state in the absence of insulin. The assumption of a constant insulin concentration is reasonable for modeling of insulin concentration given in excess. A quasi-steady-state approximation (QSSA) sets the concentration of external insulin to a constant value, $i=i_{0}$, and determines the steady states within this assumption.

**Figure 8:**
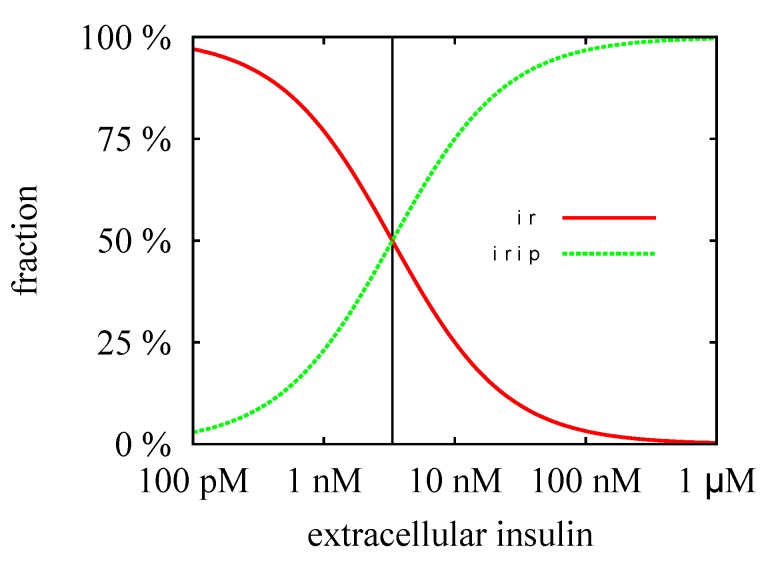
The fractions of the steady-state concentrations, $ir^{*}/ir_{0}$ and $irip^{*}/ir_{0}$, are plotted versus the concentration of the external insulin. The vertical line indicates the value of the equilibrium constant, $i_{c}=3.33$ nM. For insulin concentration above $i_{c}$, more than 50% of the extracellular IR binds an insulin molecule. All the IR becomes saturated for an insulin concentration which is large compared to $i_{c}$, *i.e.*, for $i\gg i_{c}=3.33$ nM.

The TIs decompose the network into subnetworks, for which steady states can be computed. For the subsystem defined by TI${}_{1}$, we calculated the steady-state concentrations, $ir^{*}$, $iri^{*}$, and $irip^{*}$, see Figure 4 and for the analytical formulae, see Supplemental Material. For the kinetic rate constants in Table 2, the equilibrium constant for insulin binding becomes $i_{c}=3.33$ nM. Figure 8 depicts the dependence of the fraction of accessible receptor ($ir^{*}/ir_{0}$) and activated complex ($irip^{*}/ir_{0}$) on the parameter, $i_{0}$, *i.e.*, on the concentration of extracellular insulin. The fraction, $iri^{*}/ir_{0}$, of the intermediate complex, IRI, is below 0.1 ppm and, therefore, not depicted in Figure 8. For insulin concentrations well below $i_{c}$, only a small fraction of the IR binds an insulin molecule. Increasing the concentration of external insulin, $i_{0}$, up to $i_{0}\approx i_{c}$, the steady-state concentration, $ir^{*}$, of the accessible IR drops down and simultaneously, the steady-state concentration, $irip^{*}$, of the activated complex, IRIP, grows in linear proportion to the insulin concentration $i_{0}$. Both concentrations, $ir^{*}$ and $irip^{*}$, become equal for $i_{0}=i_{c}$, see the vertical line in Figure 8. Further increase of the external insulin concentration, $i_{0}$, the steady-state concentration, $irip^{*}$, of the activated complex, IRIP, starts to predominate until nearly all the IR have been activated to IRIP.

The steady-state concentrations, $ir^{*}$, $iri^{*}$, and $irip^{*}$, describe an equilibrium of the processes, *binding*, *phosphorylation*, and *dissociation*, respectively, but completely ignore the process of translocation of activated receptor into the cytoplasm. Consequently, they represent a justifiable approximation only for a short reaction time compared to the time scale of the translocation process. The process of translocation of the activated IR into the cytoplasm ($k_{\text{in,p}}$) is member of the subnetwork, TI${}_{2}$, see Figure 5. We derived analytical expressions for the steady-state concentrations, $\;{\text{ir}}^{†},{\text{iri}}^{†},\;{\text{irip}}^{†},\;{\text{ir}}_{in}^{†},$ and $\;{\text{irip}}_{in}^{†}$, of the corresponding mass action reaction system, see Supplemental Material. The critical insulin concentration of internalization of the IR becomes $i_{c}^{†}=0.535$ nM for the kinetic rate constants in Table 2.

A substantial redistribution of the surface and intracellular IR becomes observable for external insulin concentrations around $i_{c}^{†}$. The cell is maximally down-regulated for external insulin concentrations well above $i_{c}^{†}$. The distribution of the IR in the maximally down-regulated state is approximately 59% surface and 41% intracellular. For a discussion of the experimental evidence of the distribution of the surface and intracellular IR, see the work of Quon & Campfield [31].

#### Time Behavior

Physiologically, the level of external insulin varies rapidly in response to increased blood glucose levels. The release of insulin is not continuous, but oscillates on the time scale of minutes. Due to the dynamics of the tightly regulated insulin level, the cell has no infinite time for an adaption to the external insulin level. Thus, the time scale on which the IR system will approach the quasi-steady-state concentrations is important. In the following, we discuss the dynamics of the process of down-regulation for constantly high insulin levels.

**Figure 9:**
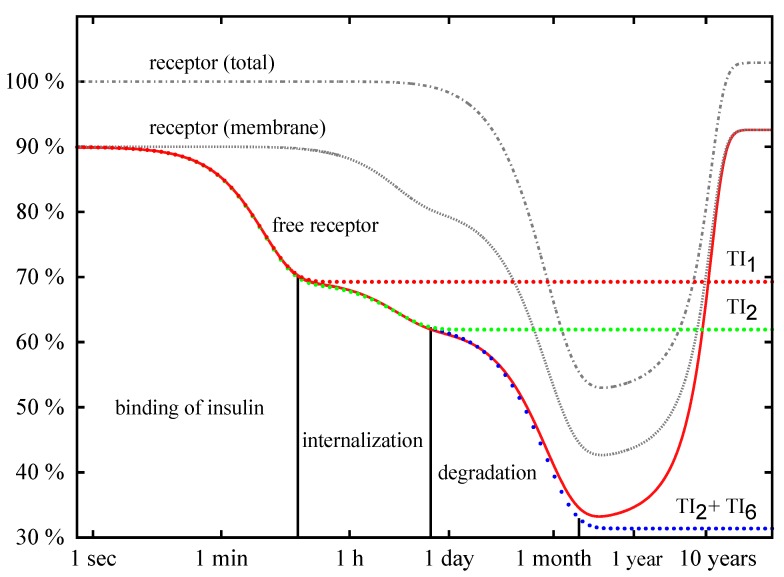
Precise numerical solutions for the concentration of accessible IR on the membrane (solid line with the label *free receptor*), the total concentration of IR on the membrane, $ir_{memb}=ir+iri+irip$ (solid line with the label *receptor (membrane)*), and the total concentration of the IR, $ir_{tot}=ir_{memb}+irip_{in}+ir_{in}$ (solid line with the label *receptor (total)*), are plotted versus the logarithmic time axis. The concentrations are given in percentage of the total concentration, $ir_{tot}$, of the IR of the basal cell. The initial insulin concentration is $i_{0}=1$ nM. The down-regulation of the cell for insulin given in excess passes through three phases: binding of insulin, internalization of the activated IR, and degradation of the IR inside the cell. The analytical approximation (Equation 6) for the concentration of the IR is drawn as dotted line with the label TI${}_{1}$ and describes the fast binding of insulin to the IR. The dotted line with the label TI${}_{2}$ shows the approximation (8). The analytical solution (Equation 11) for the concentration of the IR is depicted as dotted line with the label, TI${}_{2}$ + TI${}_{6}$, and is indistinguishable from the precise numerical solution (solid line *free receptor*) until the QSSA breaks down, *i.e.*, until the consumption of insulin becomes measurable. The concentration of insulin is not plotted.

The down-regulation of the cell for high insulin levels passes through three different phases: (1) binding of insulin to the IR; (2) internalization of the activated IR; and (3) degradation of IR in the cytoplasm. Figure 9 depicts a precise numerical solution of the complete model for the initial insulin concentration of $i_{0}=1$ nM. For the kinetic constants and the initial concentrations, see Table 1 and Table 2, respectively. The concentration of the accessible IR on the membrane (ir, broken line with the label *free receptor*), the total concentration of the IR on the membrane ($ir_{memb}=ir+iri+irip$, dotted line with the label *receptor (membrane)*), and the total concentration of the IR ($ir_{tot}=ir_{memb}+irip_{in}+ir_{in}$, solid line with the label *receptor (total)*) are plotted versus the logarithmic time axis.

In the initial state, no insulin is bound to the surface IR. The down-regulation of the cell starts with the fast binding of insulin to the IR and the activation, *i.e.*, phosphorylation, of the IR. The concentrations evolve with time towards the quasi-steady-state concentrations, $ir^{*}$ and $irip^{*}$. Analytically, the exponential functions,

(6) $$\begin{array}{ccc} ir^{\prime }\left(t\right) & = & ir^{*}+\left(ir_{0}-ir^{*}\right)\;e^{\lambda_{1}\;t}\;\text{and} \end{array}$$

(7) $$\begin{array}{ccc} irip^{\prime }\left(t\right) & = & \left(ir_{0}-ir^{*}\right)\;\left[1-e^{\lambda_{1}\;t}\right]\; \end{array}$$

describe the time course of concentrations. $\lambda_{1}$ is the characteristic eigenvalue of the ODE subsystem defined by TI${}_{1}$. For the analytical expression of $\lambda_{1}$, see Supplemental Material.

The overall rate of binding and activation depends on the initial concentration of insulin, $i_{0}$. For the initial concentrations of insulin, $i_{0}=1\;\text{nM},10\;\text{nM},100\;\text{nM},$ and 1μM, the typical scales of the reaction time are $1/\lambda_{1}=3.85\;\text{min},1.25\;\text{min},9.69\;s$, and 0.998s, respectively. The first response of the cell to an elevated level of insulin is a reorganization of the free membrane IR to the activated IRIP on the membrane.

In Figure 9, the exponential function, $ir^{\prime }\left(t\right)$, is plotted for $i_{0}=1$ nM as a dotted red line with the label TI${}_{1}$. The curve shows an initial drop and becomes constant for long time periods. During the phase of initial drop, the exponential function $ir^{\prime }\left(t\right)$ is indistinguishable from the precise numerical solution drawn as bold red line with the label *free receptor*. The left vertical line in Figure 9 indicates the position of the time point $t=3/\lambda_{1}=11\;\text{min}$. For a short reaction time (*i.e.*, t<11min), the exponential function $ir^{\prime }\left(t\right)$ correctly describes the binding of insulin to the accessible IR. On a longer time scale, $ir^{\prime }\left(t\right)$ approaches the constant value $ir^{*}$. The quasi-steady-state concentrations, $ir^{*}$, and $irip^{*}$, neglect the internalization of the phosphorylated IR, and thus, a deviation of $ir^{\prime }\left(t\right)$ from the numerical solution becomes more and more distinct for t>11min.

After the binding of insulin to the accessible IR and reaching the equilibrium concentrations, $ir^{*}$ and $irip^{*}$, the cell commences to internalize the activated IRIP. The process of internalization translocates the activated IRIP from the membrane to the cytoplasm. As a consequence, the concentration, ir, of the accessible IR as well as the total concentration, $ir_{memb}$, of the IR on the membrane drops down. The redistribution of the surface and intracellular IR does not change the total concentration, $ir_{tot}$, of the IR in the cell. Up to the end of the phase of IR internalization, the evolution of concentration with time is given by the exponential functions,

(8) $$\begin{array}{ccc} ir^{\prime \prime }\left(t\right) & = & \nu \;ir^{†}+\left(ir^{\prime }\left(t\right)-\nu \;ir^{†}\right)\;e^{\lambda_{2}\;t},\\ irip^{\prime \prime }\left(t\right) & = & \nu \;irip^{†}+\left(irip^{\prime }\left(t\right)-\nu \;irip^{†}\right)\;e^{\lambda_{2}\;t},\;\text{and}\\ ir_{in}^{\prime \prime }\left(t\right) & = & \nu \;ir_{in}^{†}+\left(ir_{in,0}-\nu \;ir_{in}^{†}\right)\;e^{\lambda_{2}\;t}\; \end{array}$$

with the eigenvalue $\lambda_{2}$ of the ODE subsystem defined by TI${}_{2}$, see Supplemental Material. The mass conservation of the IR determines the constant parameter,

(9) $$\nu :=\frac{ir_{0}+ir_{in,0}}{ir^{†}+irip^{†}+ir_{in}^{†}}=\frac{ir_{\text{tot},0}}{ir_{\text{tot}}^{†}}\;$$

with $ir_{in,0}$ as the initial (basal) concentration of the internalized IR. For the initial concentrations of insulin, $i_{0}=1\;\text{nM},10\;\text{nM},100\;\text{nM},$ and 1μM, the typical time scales of the process of receptor internalization are $1/\lambda_{2}=4\;h\;27\;\text{min}$, 3h34min, 3h18min, and3h16min, respectively. The approximation, $ir^{\prime \prime }\left(t\right)$, for the concentration, ir, of the accessible IR is plotted as a dotted line labeled by TI${}_{2}$ in Figure 9. At least for $t<3/\lambda_{2}$, the precise numerical solution for the concentration of the IR is well approximated by the superposition of two exponential functions (8); a vertical line at $t=3/\lambda_{2}=13\;h$ is drawn in Figure 9.

The process of translocation of the activated IR from the membrane into the cytoplasm leads to an accumulation of internalized IR. Note that the mass conservation,

(10) $$ir^{\prime \prime }\left(t\right)+irip^{\prime \prime }\left(t\right)+ir_{in}^{\prime \prime }\left(t\right)=ir_{0}+ir_{in,0}\;$$

is fulfilled for approximation (8), and the total amount of IR in the cell, $ir_{tot}$, does not change during the phase of internalization, see Figure 9.

In contrast to $ir_{memb}$, the level of receptor concentration in the cytoplasm grows during the phase of internalization of the membrane IR, where at the end the concentration of the IR in the cytoplasm is much higher than the steady-state value, $\;{\text{ir}}_{in}^{†}$, of receptor synthesis and degradation. Thus, a subsequent degradation of the IR down-regulates the accumulated concentration in the cytoplasm. The slow degradation of the intracellular IR leads to a decrease of concentration that can be approximated by

(11) $$\begin{array}{ccc} ir^{\prime \prime \prime }\left(t\right) & = & ir^{†}+\left(ir^{\prime \prime }\left(t\right)-ir^{†}\right)\;e^{\lambda_{3}\;t},\\ irip^{\prime \prime \prime }\left(t\right) & = & irip^{†}+\left(irip^{\prime \prime }\left(t\right)-irip^{†}\right)\;e^{\lambda_{3}\;t},\;\text{and}\;\\ ir_{in}^{\prime \prime \prime }\left(t\right) & = & ir_{in}^{†}+\left(r_{in}^{\prime \prime }\left(t\right)-ir_{in}^{†}\right)\;e^{\lambda_{3}\;t} \end{array}$$

with $\lambda_{3}=-k_{\text{syn}}/ir_{\text{tot}}^{†}$ and $ir_{\text{tot}}^{†}=ir^{†}+iri^{†}+irip^{†}+ir_{in}^{†}+irip_{in}^{†}$.

For the initial concentrations of insulin, $i_{0}=1\;\text{nM},10\;\text{nM},100\;\text{nM},$ and 1μM, the typical time scales of IR degradation are $1/\lambda_{3}=21\;\text{days},11\;\text{days},10\;\text{days}\;6\;h,$ and 10days3h, respectively. The equations (11) describe the drop of concentration of the accessible IR by three exponential phases, see $ir^{\prime \prime \prime }\left(t\right)$ plotted as dotted line with the label, TI${}_{2}+$TI${}_{6}$, in Figure 9. A deviation of $ir^{\prime \prime \prime }\left(t\right)$ from the precise numerical solution (solid line labeled *free receptor*) becomes visible only on a long time scale. The precise numerical solution does not drop down to the values of the QSSA, $ir^{†}$, $irip^{†}$, and $ir_{in}^{†}$. Instead, the concentration of the accessible receptor starts to increase again, and swings back to the concentration of the basal state. The modeled consumption of insulin by the cell leads to a decrease of external insulin, an effect that we neglect in the quasi-steady-state approximation. For exhausted external insulin, the cell turns back to the basal state.

The time scale of months makes such a consumption of insulin irrelevant for the behavior of cells in an organism that regulates tightly, *i.e.*, on the time scale of minutes the insulin level, according to a varying glucose level. At least from the academic point of view, it is interesting that the consumption of insulin described by the system is an example for the application of the Lambert function [98]. Inserting the QSSA, $ir^{†}$, $irip^{†}$, and $ir_{in}^{†}$, into the ODE system leads to

(12) $$\frac{\partial i}{\partial t}=\lambda_{4}\;\frac{i}{i_{c}^{†}+i}$$

with $\lambda_{4}=k_{\text{out}}\;k_{\text{syn}}/k_{\text{deg}}$. The analytical solution of the differential equation is given by

$$i\left(t\right)=i_{c}^{†}\;W\left(\frac{e^{\lambda_{4}\;t+C_{1}/i_{c}^{†}}}{i_{c}^{†}}\right)\;$$

where *W* stands for the Lambert function. The integration constant $C_{1}$ is determined by the initial condition $i\left(t=0\right)=i_{0}$, e.g., the initial condition, i(t=0)=1, is satisfied for $C_{1}=1$. For kinetic parameters in Table 2, we get the typical time scale of $t_{4}=1/\lambda_{4}=5\;h\;33\;\text{min}$; for a detailed discussion see Supplemental Material.

## 4. Conclusions

We applied the Petri net formalism to analyze the structural properties of a biological signaling model that describes the insulin-dependent phosphorylation and recycling of the IR. The analysis of the PN model is valuable and complementary to the numerical kinetic simulation. The concerted application of kinetic simulations and PN analysis demonstrates an advantageous strategy which is appropriate and promising also for other and larger biological systems.

First, we constructed a discrete P/T-PN model. The PN analysis started with a verification of formal constraints that a biological network has to fulfill [63]. Since the insulin model studied here is well-established in the literature, we abstain from describing the details of standard network verification. Functional modules are represented by TIs [63,99,100]. Note that the identification of functional modules in networks is a hard, computational task for a broad range of multidisciplinary applications [101,108]. Two non-trivial TI decomposed the P/T-PN into two subnetworks, defining basic functional modules, for which we determined the quasi-steady states. According to analysis of the PN model, the insulin-dependent evolution of the IR activation and recycling manifests in three phases: (1) At the initial reaction of a basal cell to an increased concentration of external insulin, the membrane IR binds insulin and becomes phosphorylated; (2) Then, internalization of the phosphorylated IR from the membrane into the cytoplasm takes place; (3) In the cytoplasm the IR is degraded. Each phase happens at a specific time scale and depicts an exponential transition from one quasi-steady state to another. Several seconds to few minutes for the binding of insulin, 3–4 h for the internalization of the IR, and 10–20 days for the intra-cellular degradation of the IR are the typical time scales for a set of standard rate constants [31,34]. The consumption of insulin resulted in a drop of insulin concentration according to the Lambert function.

Then, we constructed a timed, discrete model. The TPN model gave a rather rough approximation of the well-established mass action principle. It is surprising that the simple TPN was capable of mimicking the time behavior of the mass action kinetics to some extent. The evolution of the ODE model with time strongly relies on the dynamic equilibrium of (quasi-)steady states which are inherent properties of the mass action kinetics.

The concept of steady states is not applicable to TPNs. The TPN model achieved the desired time behavior by a completely different mechanism. The ability of a TPN model to produce a distribution of tokens that evolve in qualitative accordance with continuous concentrations of a mass action reaction system is a promising result for further studies. The values of the required time delays and the corresponding maximal token fluxes are of special interest for pharmaceutical applications. Here, a systematic mathematical strategy to learn the parameters of the TPN from experimental data would be a valuable improvement.

We demonstrated that the PN formalism enables the decomposition of the network structure into submodules and a subsequent computation of the corresponding quasi-steady states. The PN approach goes beyond previous standard steady-state analyses [20] of the Sedaghat’s model [34]. We give analytical formulas for the states and transitions inherent to the topological structure proposed. The formulas are valid for any values of kinetic rate constants or initial conditions. Hence, the analysis revealed the fundamental functional modules of dynamics that the network structure is capable to generate. The analytic formulas can easily be applied to adapt the model to experimental data, e.g., to experimental values for the IR half life and internalization rate. The presented PN approach is straightforward and worthwhile for other and more complex network structures of the IR life cycle.

**Figure 10:**
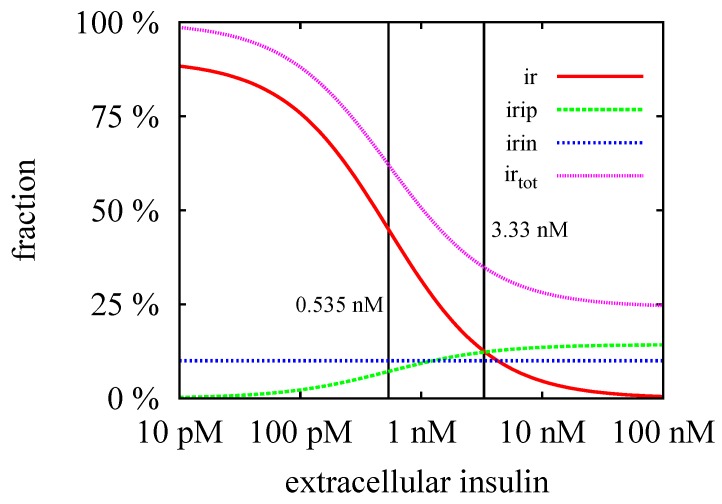
The steady-state concentrations, $ir^{†}$ (red line), $irip^{†}$ (green line), $ir_{in}^{†}$ (blue line), and $ir_{tot}=ir^{†}+irip^{†}+ir_{in}^{†}$ (violet line) are plotted versus the external insulin concentration $i_{0}$. The critical insulin concentration, $i_{c}^{†}=0.535$ nM, and the equilibrium constant, $i_{c}=3.33$ nM, are indicated by left and right vertical lines, respectively. The steady-state concentration, $ir_{in}^{†}$, is regulated by synthesis and degradation of the IR in the cytoplasm and hence, remains constant. The steady-state concentration, $irip^{†}$, is zero in the basal state of the cell, *i.e.*, in absence of extracellular insulin, $i_{0}=0$. In the process of down-regulation of the cell, *i.e.*, for increasing insulin level, $i_{0}$, the concentration, $irip^{†}$, increases until it reaches its maximal values for $i_{0}\gg i_{c}^{†}$. The steady-state concentration, $ir^{†}$, of the surface IR is maximal in the basal state and drops down to zero for $i_{0}\gg i_{c}^{†}$.

From the biological point of view, it is interesting that the PN approach models, for the first time, the experimentally well-known insulin-stimulated degradation of IR [47,72,73,74,75,76,77,78,79,80,81,82,83,84,85,86,87,88,89,90] which plays a fundamental role in the insulin resistance and metabolic disorders [55,57,59,60]. Due to the (lysosomal) IR degradation inside the cell, the total amount of the IR starts to decrease as soon as the IR becomes activated by external insulin and is transported into the cytoplasm. The IR concentration becomes significantly reduced when the system is permanently activated, see Figure 10. Note that an increase in insulin secretion characterizes prediabetic patients and type 2 diabetic patients who have developed an insulin resistance [102]. Experimentally, it is observed that the concentration of the IR is decreased to about 55% in adipocyte cells obtained from a comparison of type 2 diabetic patients and healthy adipocyte of humans [56,60]. If the reduction of the IR concentration in patients, having developed insulin resistance, is caused by a simple imbalance of degradation and synthesis of the IR within cells, it would be a promising target to inhibit the degradation of IR to treat insulin resistance in type 2 diabetic patients.

For future work, we recommend the consideration of regulatory elements for the ubiquitin-dependent degradation [55] and the transcription [54,103,104,105,106] of the IR. Such modeling approaches can give new insights into the evolution from the healthy state to metabolic disorder and can help to find strategies to reverse such an undesirable transition.

## Supplementary Materials

Supplementary materials can be accessed at: http://www.mdpi.com/2218-1989/5/4/766/s1.
