# Supplementary material for: Quasi-Steady-State Analysis based on Structural Modules and Timed Petri Net Predict System’s Dynamics: The Life Cycle of the Insulin Receptor
Source: Metabolites. 2015 Dec 18;5(4):766–93. doi: 10.3390/metabo5040766 (PMC4693194; doi:10.3390/metabo5040766)
Supplement: Supplementary file 1 [file metabolites-05-00766-s001.pdf]

Metabolites **2015**, *5*, doi:10.3390/metabo5040766

OPEN ACCESS

metabolites

ISSN 2218-1989

www.mdpi.com/journal/metabolites

Supplemental Materials

S1. List of Transition Invariants

The network has 6 transition invariants (TI):

1. TI_1: $k_{\text{bind}}, k_{\text{phos}}, k_{\text{dephos,m}}$ (binding of insulin, phosphorylation, dephosphorylation on membrane)
2. TI_2: $k_{\text{bind}}, k_{\text{phos}}, k_{\text{in,p}}, k_{\text{dephos,c}}, k_{\text{out}}$, buffer (binding of insulin, phosphorylation, internalization, cytoplasmic dephosphorylation, translocation back to membrane)
3. TI_3: $k_{\text{in}}, k_{\text{out}}$ (internalization, translocation back to membrane)
4. TI_4: $k_{\text{in,p}}, k_{\text{out,p}}$ (internalization of phosphorylated insulin receptor (IR), translocation back to membrane)
5. TI_5: $k_{\text{bind}}, k_{\text{diss}}$ (extracellular binding of insulin, release of insulin)
6. TI_6: $k_{\text{syn}}, k_{\text{deg}}$ (synthesis, degradation of receptor)

Each transition is member of at least one TI, hence the network is covered by TI (CTI).

S2. Quasi-Steady-State Approximation for TI_1

TI_1 describes a cycle of reactions for the species IR, IRI, and IRIP. The corresponding dynamic system is given by

$$\frac{\partial \vec{c}}{\partial t} = \begin{pmatrix} -k_{\text{bind}}\, i_0 & k_{\text{diss}} & k_{\text{dephos,m}} \\ +k_{\text{bind}}\, i_0 & -k_{\text{diss}} - k_{\text{phos}} & 0 \\ 0 & k_{\text{phos}} & -k_{\text{dephos,m}} \end{pmatrix} \vec{c}\,, \tag{S1}$$

where $\vec{c} = (\text{ ir}, \text{iri}, \text{ irip})^T$ denotes a vector of concentrations. The concentration of free insulin is assumed to be constant, *i.e.*, $i = i_0$. Within the QSSA we solved the linear system

$$\frac{\partial \vec{c}}{\partial \tau} = 0 \tag{S2}$$

and obtained the steady state for the 3 concentrations

$$\begin{aligned} ir^* &= \left[1 - \frac{i_0}{i_0 + i_c}\right] ir_0\,, \\ iri^* &= \frac{k_{\text{dephos,m}}}{k_{\text{phos}}} \left(1 + \frac{k_{\text{dephos,m}}}{k_{\text{phos}}}\right)^{-1} \frac{i_0}{i_0 + i_c}\, ir_0\,, \text{ and} \\ irip^* &= \left[1 - \frac{k_{\text{dephos,m}}}{k_{\text{phos}}} \left(1 + \frac{k_{\text{dephos,m}}}{k_{\text{phos}}}\right)^{-1}\right] \frac{i_0}{i_0 + i_c}\, ir_0 \end{aligned} \tag{S3}$$

with the equilibrium constant

$$i_c = \frac{k_{\text{dephos,m}}}{k_{\text{bind}}} \left(1 + \frac{k_{\text{diss}}}{k_{\text{phos}}}\right) \left(1 + \frac{k_{\text{dephos,m}}}{k_{\text{phos}}}\right)^{-1} . \tag{S4}$$

For our choice of kinetic rate constants, the insulin-binding equilibrium constant becomes $i_c = 3.33$ nM. Sedaghat *et al.* assume a fast process of phosphorylation (*i.e.*, $k_{\text{phos}} \gg k_{\text{diss}}$ and $k_{\text{phos}} \gg k_{\text{dephos,m}}$). In this case the equation

$$i_c \approx \frac{k_{\text{dephos,m}}}{k_{\text{bind}}} \tag{S5}$$

is a reasonable approximation. Since the ratio $k_{\text{dephos,m}}/k_{\text{phos}}$ is less than 0.1 %, we may neglect iri^*, and the formula

$$irip^* \approx \frac{i_0}{i_0 + i_c}\, ir_0$$

is sufficiently precise for practical applications.

S3. Quasi-Steady-State Approximation for TI$_2$

The steady-state concentrations ir^*, iri^*, and $irip^*$ completely ignore the process of translocation of receptor into the cytoplasm and are a justifiable approximation only for a short reaction time compared to the time scale of the translocation process. The process of translocation of the activated IR into the cytoplasm ($k_{\text{in,p}}$) is member of the subnetwork defined by TI$_2$. The ODE system of the subnetwork reads

$$\frac{\partial \vec{c}}{\partial t} = \begin{pmatrix} 0 \\ 0 \\ 0 \\ k_{\text{syn}} \\ 0 \end{pmatrix} - \begin{pmatrix} k_{\text{bind}}\, i_0 + k_{\text{in}} & -k_{\text{diss}} & -k_{\text{dephos,m}} & -k_{\text{out}} & 0 \\ -k_{\text{bind}}\, i_0 & k_{\text{diss}} + k_{\text{phos}} & 0 & 0 & 0 \\ 0 & -k_{\text{phos}} & k_{\text{dephos,m}} + k_{\text{in,p}} & 0 & -k_{\text{out,p}} \\ -k_{\text{in}} & 0 & 0 & k_{\text{out}} + k_{\text{deg}} & -k_{\text{dephos,c}} \\ 0 & 0 & -k_{\text{in,p}} & 0 & k_{\text{dephos,c}} + k_{\text{out,p}} \end{pmatrix} \vec{c} \tag{S6}$$

with the vector of concentrations, $\vec{c} = (\text{ ir}, \text{iri}, \text{ irip}, \text{ ir}_{in}, \text{ irip}_{in})^T$. The steady state is given by

$$\begin{aligned}
ir^\dagger &= \frac{i_0}{i_c^\dagger + i_0} \left(1 + \frac{k_{\text{diss}}}{k_{\text{phos}}}\right) \left[\frac{k_{\text{dephos,m}}(k_{\text{out,p}} + k_{\text{dephos,c}})}{k_{\text{bind}} i_0\, k_{\text{in,p}}} + \frac{k_{\text{dephos,c}}}{k_{\text{bind}} i_0}\right] \frac{k_{\text{out}}}{k_{\text{dephos,c}}}\, ir_{in}^\dagger , \\
iri^\dagger &= \frac{i_0}{i_c^\dagger + i_0} \left[\frac{k_{\text{dephos,m}}(k_{\text{out,p}} + k_{\text{dephos,c}})}{k_{\text{phos}} k_{\text{in,p}}} + \frac{k_{\text{dephos,c}}}{k_{\text{phos}}}\right] \frac{k_{\text{out}}}{k_{\text{dephos,c}}}\, ir_{in}^\dagger , \\
irip^\dagger &= \frac{i_0}{i_c^\dagger + i_0} \frac{k_{\text{out,p}} + k_{\text{dephos,c}}}{k_{\text{in,p}}} \frac{k_{\text{out}}}{k_{\text{dephos,c}}}\, ir_{in}^\dagger , \\
ir_{in}^\dagger &= \frac{k_{\text{syn}}}{k_{\text{deg}}} , \text{ and} \\
irip_{in}^\dagger &= \frac{i_0}{i_c^\dagger + i_0} \frac{k_{\text{out}}}{k_{\text{dephos,c}}}\, ir_{in}^\dagger
\end{aligned} \tag{S7}$$

with the constant

$$i_c^\dagger = \frac{k_{\text{in}}}{k_{\text{bind}}} \left[1 + \frac{k_{\text{dephos,m}}}{k_{\text{in,p}}} \left(1 + \frac{k_{\text{out,p}}}{k_{\text{dephos,c}}}\right)\right] \left(1 + \frac{k_{\text{diss}}}{k_{\text{phos}}}\right) . \tag{S8}$$

$i_c^\dagger$ is the critical insulin concentration for the internalization of receptor. For a fast phosphorylation process as postulated by Sedaghat *et al.*, (*i.e.*, $k_{\text{phos}} = 2.500\ \text{min}^{-1}$) a simplification of equations (S7,S8) is feasible.

We considered nonzero degradation and nonzero synthesis of the receptor, *i.e.*, k_{syn}, k_{deg}, in the steady state (S7). However, the degradation and synthesis are not members of TI_2 but form the trivial TI_6. For $k_{\text{syn}} = k_{\text{deg}} = 0$ (*i.e.* in the case of no degradation and no synthesis), the steady-state concentration, $ir_{in}^\dagger$, becomes a free parameter and has to be determined by a mass conservation equation for the amount of the receptor in the cell.

For our choice of kinetic constants, we get the numerical value, $i_c^\dagger = 0.535$ nM, for the critical insulin concentration of internalization of the IR and the steady state concentrations (S7) become

$$\begin{aligned} ir^\dagger &= 0.9\ \text{pM} \times \left[1 - \frac{i_0}{i_c^\dagger + i_0}\right] , \\ iri^\dagger &= 0.0116\ \text{fM} \times \frac{i_0}{i_c^\dagger + i_0} , \\ irip^\dagger &= 0.143\ \text{pM} \times \frac{i_0}{i_c^\dagger + i_0} , \\ ir_{in}^\dagger &= 0.1\ \text{pM} , \text{ and} \\ irip_{in}^\dagger &= 0.651\ \text{fM} \times \frac{i_0}{i_c^\dagger + i_0} . \end{aligned} \tag{S9}$$

The steady state concentrations, $iri^\dagger$ and $irip_{in}^\dagger$, of the transient complexes are below experimental detection limits. The steady state concentration, $ir_{in}^\dagger$, of free intracellular receptor is regulated by synthesis (k_{syn}) and degradation (k_{deg}), and hence remains constant for all values of i_0. In the limit of small concentrations of insulin, $i_0 \to 0$, the function

$$f(i_0) = \frac{i_0}{i_c^\dagger + i_0} \tag{S10}$$

approaches zero for vanishing concentration of external insulin, *i.e.*, $\lim_{i_0 \to 0} f(i_0) = 0$. For increasing concentrations of insulin, $i_0 \to \infty$, the function $f(i_0)$ converges to 1. Since the steady-state concentrations, $iri^\dagger$, $irip^\dagger$ and $irip_{in}^\dagger$, are proportional to $f(i_0)$, they are zero in the basal state of the cell, *i.e.*, in absence of extracellular insulin, $i_0 = 0$. In the process of down-regulation by insulin, the concentrations, $iri^\dagger$, $irip^\dagger$, and $irip_{in}^\dagger$, increase proportionally to the function $f(i_0)$ until they reach their maximal values for $i_0 \gg i_c^\dagger$. The steady-state concentration, $ir^\dagger$, of the surface receptor is proportional to $1 - f(i_0)$, and hence, $ir^\dagger$ is maximal in the basal state and drops down to zero for $i_0 \gg i_c^\dagger$.

S4. Characteristic Eigenvalue for TI_1

The characteristic eigenvalue of ODE (S1) is given by

$$\lambda_1 = -\frac{k_{\text{bind}}\, i_0 + k_{\text{diss}} + k_{\text{phos}} + k_{\text{dephos,m}}}{2} \left[1 - \sqrt{1 - \frac{4(k_{\text{bind}}\, i_0\, (k_{\text{phos}} + k_{\text{dephos,m}}) + (k_{\text{diss}} + k_{\text{phos}})\, k_{\text{dephos,m}})}{(k_{\text{bind}}\, i_0 + k_{\text{diss}} + k_{\text{phos}} + k_{\text{dephos,m}})^2}}\right] . \tag{S11}$$

The simplification

$$\lambda_1 \approx -\frac{k_{\text{phos}}\,(k_{\text{bind}}\,i_0 + k_{\text{dephos,m}})}{k_{\text{bind}}\,i_0 + k_{\text{phos}}} \tag{S12}$$

approximates the eigenvalue, λ_1, within a relative precision of 2×10^{-5}.

S5. Characteristic Eigenvalue for TI$_2$

The characteristic eigenvalue of ODE (S6) is given by

$$\begin{aligned}
\lambda_2 &= -\frac{L}{2}\left[1-\sqrt{1-\frac{4(k_{\text{out}}(k_{\text{out,p}}+k_{\text{dephos,c}})+(k_{\text{out,p}}+k_{\text{dephos,c}})K_1+(k_{\text{out}}+k_{\text{dephos,c}})K_2)}{L^2}}\,\right],\\
L &= k_{\text{out,p}}+k_{\text{out}}+k_{\text{dephos,c}}+K_1+K_2,\\
K_1 &= k_{\text{in}}\,\frac{i_c}{i_0+i_c},\ \text{and}\\
K_2 &= \frac{k_{\text{phos}}k_{\text{in,p}}}{k_{\text{phos}}+k_{\text{dephos,m}}}\,\frac{i_0}{i_0+i_c}\quad .
\end{aligned}$$

S6. Drop of Insulin and the Lambert Function

We have abstained from discussing the development of insulin concentration with time based on the functional regimes of the Lambert function *W*. It is easy to see that for insulin concentrations well below the critical concentration of $i_c^\dagger = 0.535$ nM, the differential equation simplifies to

$$\frac{\partial\,\text{i}}{\partial t} = -\frac{\text{i}}{t_4}\,, \tag{S13}$$

and the insulin concentration drops down exponentially in time

$$i(t) = i_0\, e^{-t/t_4}\,. \tag{S14}$$

In the case of a high concentration of insulin (*i.e.*, for $i \gg i_c^\dagger = 0.535$ nM), the cell is maximally down-regulated, and the differential equation is given by

$$\frac{\partial\,\text{i}}{\partial t} = -\frac{i_c}{t_4}\,. \tag{S15}$$

Consequently, the consumption of insulin with constant maximal velocity leads to a linear diminishment of insulin:

$$i(t) = i_0 - i_c\,\frac{t}{t_4}\,. \tag{S16}$$

The consumption of insulin by the cell leads to an exponential drop on the time scale of $t_4 = 5\,\text{h}\;33\,\text{min}$, if the insulin concentration is below the critical insulin concentration, $i_c^\dagger = 0.535$ nM. For insulin given in excess (*i.e.*, for $i \gg i_c^\dagger$), the insulin concentration decreases linearly with a flat-angle slope of $0.535\ \text{nM}/5\,\text{h}\;33\,\text{min}$.

S7. Phosphorylation Dynamics

Cedersund *et. al.* [1] have discussed the short-term phosphorylation dynamics of the insulin receptor. They have measured a rapid transient overshoot in tyrosine phosphorylation for human adipocytes after a step increase from 0 to 0.1 μM in insulin concentration and have discussed the implication of such an "overshot" on various model structures. Cedersund *et. al.* [2] have rejected model structures based on the zeros and complex poles of the linearized transfer function, see also Brännmark *et. al.* [3]. In terms of the Petri net formalism, the model structure requires a certain substructure to produce an overshot behavior. For Sedaghat *et al.*'s model [4] such a substructure is defined by transition invariant TI_1. The Petri net approach explains the overshot by the high concentration of phosphorylated receptor, $irip^*$, of the meta-stable quasi-steady state associated with transition invariant TI_1. Figure S1 shows the percentage of transient phosphorylated IR versus the concentration of insulin.

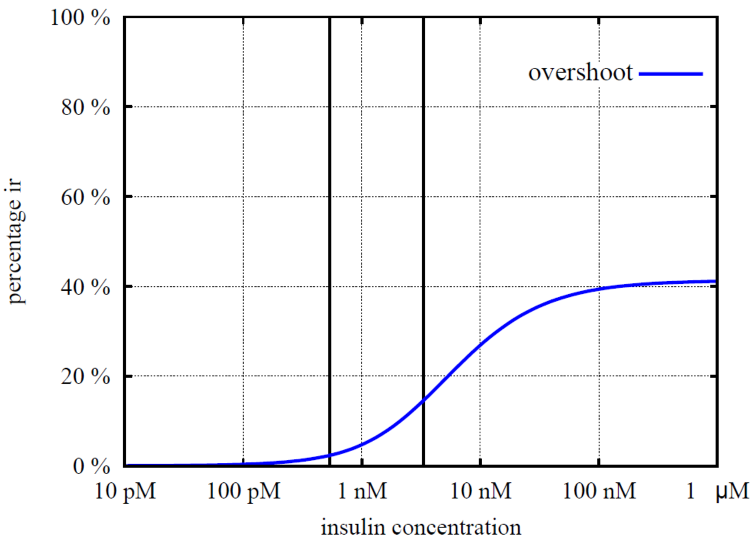

Figure S1. After a step increase in insulin concentration the concentration of phosphorylated IR approach the value $irip^*$ of meta-stable steady state (S3). This transient high value of phosphorylated IR drops to the value $\nu \times irip^\dagger$ of meta-stable steady state (S7) due to endocytosis and dephosphorylation of the internalized IR. Plotted is the percentage of transient phosphorylated $irip^* - \nu \times irip^\dagger$ versus the concentration of insulin. For 0.1 μM insulin concentration, Sedaghat *et al.*'s model estimates an "overshoot" at in round numbers 40% .

References

1. Cedersund, G.; Roll, J.; Ulfhielm, E.; Danielsson, A.; Tidefelt, H.; Strålfors P. Model-based hypothesis testing of key mechanisms in initial phase of insulin signaling. *PLoS Comput. Biol.* **2008**, *4*, e1000096.
2. Cedersund, G.; Roll, J. Systems biology: Model based evaluation and comparison of potential explanations for given biological data. *FEBS J.* **2009**, *276*, 903–922.

3. Brännmark, C.; Palmér R.; Glad, S.T.; Cedersund, G.; Strålfors P. Mass and information feedbacks through receptor endocytosis govern insulin signaling as revealed using a parameter-free modeling framework. *J. Biol. Chem.* **2010**, *285*, 20171–20179.
4. Sedaghat, A.R.; Sherman, A.; Quon, M.J. A mathematical model of metabolic insulin signaling pathways. *Am. J. Physiol. Endocrinol. Metabol.* **2002**, *283*, E1084–E1101.

© 2015 by the authors; licensee MDPI, Basel, Switzerland. This article is an open access article distributed under the terms and conditions of the Creative Commons Attribution license (http://creativecommons.org/licenses/by/4.0/).
